# 3-Benzyl-9-phenyl-2-tosyl-2,3,3a,4,9,9a-hexa­hydro-1*H*-pyrrolo[3,4-*b*]quinoline

**DOI:** 10.1107/S1600536809044547

**Published:** 2009-10-31

**Authors:** K. Chinnakali, D. Sudha, M. Jayagobi, R. Raghunathan, Hoong-Kun Fun

**Affiliations:** a Department of Physics, Anna University Chennai, Chennai 600 025, India; b Department of Organic Chemistry, University of Madras, Guindy Campus, Chennai 600 025, India; c X-ray Crystallography Unit, School of Physics, Universiti Sains Malaysia, 11800 USM, Penang, Malaysia.

## Abstract

In the title compound, C_31_H_30_N_2_O_2_S, the pyrrolidine ring adopts a twist conformation while the tetra­hydro­pyridine ring is in a half-chair conformation. The two rings are *trans*-fused. The pyridine-bound phenyl ring forms dihedral angles of 17.7 (1) and 48.1 (1)°, respectively, with the tosyl and benzyl phenyl rings. The mol­ecular structure is stabilized by an N—H⋯π inter­action involving the benzyl phenyl ring. In the crystal structure, mol­ecules translated by one unit along the *a* axis are linked into chains by C—H⋯π inter­actions involving the benzene ring of the tosyl group.

## Related literature

For the biological activity of pyrroloquinoline derivatives, see: Ferlin *et al.* (2005 ▶); Dalla Via *et al.* (2008 ▶); Xiao *et al.* (2006 ▶); Fujita *et al.* (1996 ▶); Crenshaw *et al.* (1976 ▶). For the crystal structure of the 3-ethyl analogue, see: Sudha *et al.* (2008 ▶). For ring puckering parameters, see: Cremer & Pople (1975 ▶). For asymmetry parameters, see: Duax *et al.* (1976 ▶).
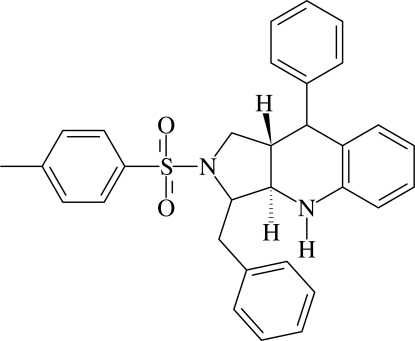


## Experimental

### 

#### Crystal data

C_31_H_30_N_2_O_2_S
*M* *_r_* = 494.63
Triclinic, 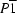

*a* = 10.9521 (3) Å
*b* = 11.2563 (3) Å
*c* = 12.5132 (3) Å
α = 100.930 (2)°
β = 108.577 (1)°
γ = 114.539 (1)°
*V* = 1234.00 (6) Å^3^
*Z* = 2
Mo *K*α radiation
μ = 0.16 mm^−1^
*T* = 100 K
0.48 × 0.24 × 0.23 mm

#### Data collection

Bruker SMART APEXII CCD area-detector diffractometer
Absorption correction: multi-scan (*SADABS*; Bruker, 2005 ▶) *T*_min_ = 0.761, *T*_max_ = 0.963
27773 measured reflections
6486 independent reflections
5001 reflections with *I* > 2σ(*I*)
*R*_int_ = 0.040

#### Refinement

*R*[*F* ^2^ > 2σ(*F* ^2^)] = 0.045
*wR*(*F* ^2^) = 0.112
*S* = 1.01
6486 reflections
330 parameters
H atoms treated by a mixture of independent and constrained refinement
Δρ_max_ = 0.51 e Å^−3^
Δρ_min_ = −0.42 e Å^−3^

### 

Data collection: *APEX2* (Bruker, 2005 ▶); cell refinement: *SAINT* (Bruker, 2005 ▶); data reduction: *SAINT*; program(s) used to solve structure: *SHELXTL* (Sheldrick, 2008 ▶); program(s) used to refine structure: *SHELXTL*; molecular graphics: *SHELXTL*; software used to prepare material for publication: *SHELXTL* and *PLATON* (Spek, 2009 ▶).

## Supplementary Material

Crystal structure: contains datablocks global, I. DOI: 10.1107/S1600536809044547/wn2361sup1.cif

Structure factors: contains datablocks I. DOI: 10.1107/S1600536809044547/wn2361Isup2.hkl

Additional supplementary materials:  crystallographic information; 3D view; checkCIF report

## Figures and Tables

**Table 1:** Hydrogen-bond geometry (Å, °)

*D*—H⋯*A*	*D*—H	H⋯*A*	*D*⋯*A*	*D*—H⋯*A*
N2—H1*N*2⋯*Cg*2	0.83 (2)	2.61 (2)	3.374 (2)	152 (2)
C29—H29⋯*Cg*1^i^	0.93	2.90	3.605 (2)	134

Symmetry code: (i) 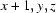. *Cg*1 and *Cg*2 are the centroids of the C19–C24 and C26–C31 rings, respectively.

**Fig. 1.**
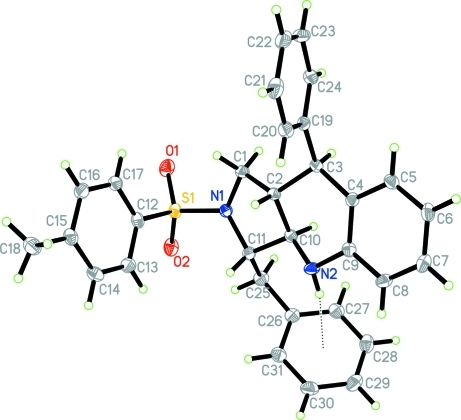
The molecular structure of the title compound. Displacement ellipsoids are drawn at the 50% probability level. Hydrogen atoms are shown as spheres of arbitrary radius. The dotted line indicates an N—H···π interaction.

**Fig. 2.**
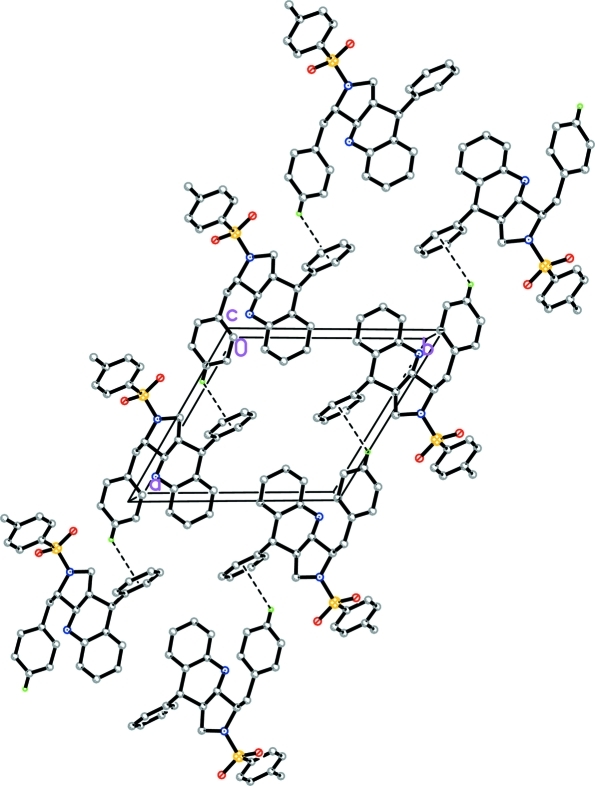
Crystal packing of the title compound. C—H···π interactions are shown as dashed lines. For the sake of clarity, H atoms not involved in these interactions have been omitted.

## supplementary crystallographic information

### Comment

Pyrroloquinoline derivatives exhibit antitumor (Ferlin *et al.*, 2005; Dalla Via *et al.*, 2008), cytotoxic (Xiao *et al.*, 2006), antibacterial (Fujita *et al.*, 1996) and interferon-inducing activities (Crenshaw *et al.*, 1976). As part of our studies on pyrroloquinoline derivatives, we report here the crystal structure of the title compound.

In the title molecule, the pyrrolidine ring adopts a twist conformation; the asymmetry parameter ΔC_2_[C2—C10] (Duax *et al.*, 1976) and the puckering parameters q_2_ and φ (Cremer & Pople, 1975) are 7.5 (2)°, 0.394 (2) Å and 96.5 (3)°, respectively. The tosyl group is attached to the pyrrolidine ring in an equatorial position. The tetrahydropyridine ring adopts a half-chair conformation, with Q, θ, φ and ΔC_2_[C4—C9] values of 0.543 (2) Å, 131.1 (2)°, 93.1 (3)° and 6.6 (2)°, respectively. The phenyl group is attached to the tetrahydropyridine ring in a biaxial position. The C19—C24 phenyl ring forms dihedral angles of 74.0 (1) and 17.7 (1)°, respectively, with the C4—C9 and C12—C17 benzene rings. The C12—C17 and C26—C31 rings are oriented at a dihedral angle of 48.1 (1)°. The molecular structure is stabilized by an N—H···π interaction (Table 1, Fig. 1). Bond lengths and angles are comparable to those observed in the 3-ethyl analogue (Sudha *et al.*, 2008).

In the crystal structure, molecules translated by one unit along the *a* axis are linked into chains by C—H···π interactions (Fig.2, Table 1) involving the benzene ring of the tosyl group.

### Experimental

InCl_3_ (20 mol%) was added to a mixture of 2-(*N*-cinnamyl-*N*-tosylamino)-3-phenyl propanal (1 mmol) and aniline (1 mmol) in acetonitrile (20 ml). The reaction mixture was stirred at room temperature for 1 min. On completion of the reaction, as indicated by TLC, the mixture was quenched with water and extracted with ethyl acetate. The organic layer was washed with brine and dried over Na_2_SO_4_. The solvent was evaporated *in vacuo* and the crude product was chromatographed on silica gel using a hexane-ethyl acetate (8.5:1.5 *v*/*v*) mixture to obtain the title compound. The compound was recrystallized from ethyl acetate solution by slow evaporation.

### Refinement

The N-bound H atom was located from a difference map and refined freely. The remaining H atoms were positioned geometrically (C—H = 0.93–0.98 Å) and allowed to ride on their parent atoms, with *U*_iso_(H) = 1.2*U*_eq_(C) or 1.5*U*_eq_(C_methyl_). A rotating group model was used for the methyl groups.

### Crystal data

**Table:**

C_31_H_30_N_2_O_2_S	*Z* = 2
*M**_r_* = 494.63	*F*(000) = 524
Triclinic, *P*1	*D*_x_ = 1.331 Mg m^−^^3^
Hall symbol: -P 1	Mo *K*α radiation, λ = 0.71073 Å
*a* = 10.9521 (3) Å	Cell parameters from 9232 reflections
*b* = 11.2563 (3) Å	θ = 2.3–30.0°
*c* = 12.5132 (3) Å	µ = 0.16 mm^−^^1^
α = 100.930 (2)°	*T* = 100 K
β = 108.577 (1)°	Block, colourless
γ = 114.539 (1)°	0.48 × 0.24 × 0.23 mm
*V* = 1234.00 (6) Å^3^	

### Data collection

**Table:**

Bruker SMART APEXII CCD area-detector diffractometer	6486 independent reflections
Radiation source: fine-focus sealed tube	5001 reflections with *I* > 2σ(*I*)
graphite	*R*_int_ = 0.040
φ and ω scans	θ_max_ = 29.0°, θ_min_ = 2.1°
Absorption correction: multi-scan (*SADABS*; Bruker, 2005)	*h* = −14→14
*T*_min_ = 0.761, *T*_max_ = 0.963	*k* = −15→15
27773 measured reflections	*l* = −17→17

### Refinement

**Table:**

Refinement on *F*^2^	Primary atom site location: structure-invariant direct methods
Least-squares matrix: full	Secondary atom site location: difference Fourier map
*R*[*F*^2^ > 2σ(*F*^2^)] = 0.045	Hydrogen site location: inferred from neighbouring sites
*wR*(*F*^2^) = 0.112	H atoms treated by a mixture of independent and constrained refinement
*S* = 1.01	*w* = 1/[σ^2^(*F*_o_^2^) + (0.046*P*)^2^ + 0.7475*P*] where *P* = (*F*_o_^2^ + 2*F*_c_^2^)/3
6486 reflections	(Δ/σ)_max_ = 0.001
330 parameters	Δρ_max_ = 0.51 e Å^−^^3^
0 restraints	Δρ_min_ = −0.42 e Å^−^^3^

### Special details

**Table:**

Geometry. All e.s.d.'s (except the e.s.d. in the dihedral angle between two l.s. planes) are estimated using the full covariance matrix. The cell e.s.d.'s are taken into account individually in the estimation of e.s.d.'s in distances, angles and torsion angles; correlations between e.s.d.'s in cell parameters are only used when they are defined by crystal symmetry. An approximate (isotropic) treatment of cell e.s.d.'s is used for estimating e.s.d.'s involving l.s. planes.
Refinement. Refinement of *F*^2^ against ALL reflections. The weighted *R*-factor *wR* and goodness of fit *S* are based on *F*^2^, conventional *R*-factors *R* are based on *F*, with *F* set to zero for negative *F*^2^. The threshold expression of *F*^2^ > σ(*F*^2^) is used only for calculating *R*-factors(gt) *etc*. and is not relevant to the choice of reflections for refinement. *R*-factors based on *F*^2^ are statistically about twice as large as those based on *F*, and *R*- factors based on ALL data will be even larger.

### Fractional atomic coordinates and isotropic or equivalent isotropic displacement parameters (Å^2^)

**Table:**

	*x*	*y*	*z*	*U*_iso_*/*U*_eq_	
S1	0.36310 (4)	0.70596 (4)	−0.01644 (4)	0.01857 (10)	
O1	0.25363 (13)	0.74003 (13)	−0.07610 (11)	0.0250 (3)	
O2	0.40078 (13)	0.62325 (12)	−0.08784 (10)	0.0240 (3)	
N1	0.51710 (14)	0.85539 (13)	0.07238 (12)	0.0173 (3)	
N2	0.87750 (15)	1.02866 (14)	0.33742 (12)	0.0179 (3)	
H1N2	0.939 (2)	1.009 (2)	0.3253 (18)	0.026 (5)*	
C1	0.50851 (17)	0.96845 (16)	0.14938 (14)	0.0181 (3)	
H1A	0.4181	0.9292	0.1604	0.022*	
H1B	0.5109	1.0381	0.1136	0.022*	
C2	0.64811 (17)	1.03222 (15)	0.26952 (14)	0.0150 (3)	
H2	0.6244	0.9770	0.3194	0.018*	
C3	0.72246 (17)	1.18825 (15)	0.34620 (14)	0.0151 (3)	
H3	0.7343	1.2408	0.2925	0.018*	
C4	0.87963 (17)	1.23749 (16)	0.44241 (14)	0.0158 (3)	
C5	0.95793 (18)	1.36418 (16)	0.54193 (15)	0.0185 (3)	
H5	0.9122	1.4167	0.5496	0.022*	
C6	1.10111 (18)	1.41433 (17)	0.62954 (15)	0.0210 (3)	
H6	1.1509	1.4995	0.6945	0.025*	
C7	1.16958 (18)	1.33567 (17)	0.61916 (15)	0.0215 (3)	
H7	1.2659	1.3683	0.6774	0.026*	
C8	1.09513 (18)	1.20931 (17)	0.52270 (15)	0.0194 (3)	
H8	1.1414	1.1570	0.5169	0.023*	
C9	0.95044 (17)	1.15885 (16)	0.43336 (14)	0.0165 (3)	
C10	0.75726 (17)	1.00918 (16)	0.23056 (14)	0.0156 (3)	
H10	0.7982	1.0779	0.1954	0.019*	
C11	0.66205 (17)	0.86091 (16)	0.13260 (14)	0.0171 (3)	
H11	0.6487	0.7906	0.1703	0.021*	
C12	0.30055 (17)	0.61554 (16)	0.07342 (14)	0.0183 (3)	
C13	0.35945 (18)	0.53371 (16)	0.11236 (15)	0.0207 (3)	
H13	0.4332	0.5291	0.0933	0.025*	
C14	0.30604 (19)	0.45969 (17)	0.17978 (15)	0.0218 (3)	
H14	0.3452	0.4057	0.2060	0.026*	
C15	0.19503 (18)	0.46425 (16)	0.20920 (15)	0.0202 (3)	
C16	0.13799 (18)	0.54575 (17)	0.16856 (15)	0.0217 (3)	
H16	0.0632	0.5492	0.1866	0.026*	
C17	0.19011 (18)	0.62183 (16)	0.10184 (15)	0.0208 (3)	
H17	0.1515	0.6765	0.0764	0.025*	
C18	0.1399 (2)	0.38679 (18)	0.28517 (17)	0.0254 (4)	
H18A	0.0355	0.3553	0.2593	0.038*	
H18B	0.1958	0.4487	0.3691	0.038*	
H18C	0.1533	0.3070	0.2755	0.038*	
C19	0.63082 (17)	1.21998 (16)	0.40329 (14)	0.0164 (3)	
C20	0.61254 (18)	1.17490 (17)	0.49677 (15)	0.0205 (3)	
H20	0.6525	1.1206	0.5211	0.025*	
C21	0.53582 (19)	1.20987 (18)	0.55365 (16)	0.0254 (4)	
H21	0.5250	1.1796	0.6160	0.031*	
C22	0.47489 (19)	1.29029 (18)	0.51758 (17)	0.0270 (4)	
H22	0.4227	1.3134	0.5553	0.032*	
C23	0.49216 (19)	1.33563 (18)	0.42575 (16)	0.0248 (4)	
H23	0.4523	1.3902	0.4021	0.030*	
C24	0.56895 (18)	1.30027 (16)	0.36793 (15)	0.0200 (3)	
H24	0.5789	1.3305	0.3054	0.024*	
C25	0.72722 (18)	0.83906 (18)	0.04379 (15)	0.0213 (3)	
H25A	0.6672	0.7416	−0.0120	0.026*	
H25B	0.7235	0.8977	−0.0034	0.026*	
C26	0.88731 (18)	0.87601 (17)	0.11140 (14)	0.0193 (3)	
C27	1.00564 (19)	1.00373 (17)	0.12935 (15)	0.0219 (3)	
H27	0.9855	1.0633	0.0942	0.026*	
C28	1.1526 (2)	1.04302 (18)	0.19876 (16)	0.0251 (4)	
H28	1.2304	1.1272	0.2077	0.030*	
C29	1.18447 (19)	0.95779 (19)	0.25491 (16)	0.0262 (4)	
H29	1.2833	0.9853	0.3030	0.031*	
C30	1.0679 (2)	0.83100 (19)	0.23886 (17)	0.0259 (4)	
H30	1.0886	0.7739	0.2773	0.031*	
C31	0.92062 (19)	0.78886 (17)	0.16586 (16)	0.0226 (4)	
H31	0.8434	0.7019	0.1531	0.027*	

### Atomic displacement parameters (Å^2^)

**Table:**

	*U*^11^	*U*^22^	*U*^33^	*U*^12^	*U*^13^	*U*^23^
S1	0.01624 (19)	0.01639 (19)	0.01461 (19)	0.00506 (15)	0.00412 (15)	0.00178 (14)
O1	0.0177 (6)	0.0274 (6)	0.0207 (6)	0.0083 (5)	0.0026 (5)	0.0090 (5)
O2	0.0232 (6)	0.0204 (6)	0.0171 (6)	0.0060 (5)	0.0082 (5)	−0.0016 (5)
N1	0.0150 (6)	0.0137 (6)	0.0176 (7)	0.0066 (5)	0.0048 (5)	0.0009 (5)
N2	0.0174 (7)	0.0189 (7)	0.0163 (7)	0.0124 (6)	0.0047 (5)	0.0017 (5)
C1	0.0167 (7)	0.0165 (7)	0.0197 (8)	0.0095 (6)	0.0072 (6)	0.0033 (6)
C2	0.0156 (7)	0.0141 (7)	0.0158 (7)	0.0083 (6)	0.0074 (6)	0.0043 (6)
C3	0.0156 (7)	0.0134 (7)	0.0160 (7)	0.0076 (6)	0.0070 (6)	0.0042 (6)
C4	0.0149 (7)	0.0154 (7)	0.0166 (7)	0.0069 (6)	0.0077 (6)	0.0053 (6)
C5	0.0200 (8)	0.0152 (7)	0.0207 (8)	0.0091 (6)	0.0106 (6)	0.0046 (6)
C6	0.0179 (8)	0.0163 (7)	0.0188 (8)	0.0042 (6)	0.0062 (6)	0.0012 (6)
C7	0.0146 (7)	0.0235 (8)	0.0186 (8)	0.0064 (6)	0.0052 (6)	0.0042 (7)
C8	0.0178 (8)	0.0227 (8)	0.0206 (8)	0.0129 (7)	0.0088 (6)	0.0074 (7)
C9	0.0172 (7)	0.0164 (7)	0.0158 (7)	0.0081 (6)	0.0085 (6)	0.0048 (6)
C10	0.0157 (7)	0.0144 (7)	0.0154 (7)	0.0078 (6)	0.0064 (6)	0.0035 (6)
C11	0.0153 (7)	0.0154 (7)	0.0164 (7)	0.0074 (6)	0.0049 (6)	0.0022 (6)
C12	0.0179 (8)	0.0135 (7)	0.0148 (7)	0.0045 (6)	0.0047 (6)	0.0011 (6)
C13	0.0203 (8)	0.0175 (7)	0.0209 (8)	0.0094 (7)	0.0091 (7)	0.0016 (6)
C14	0.0270 (9)	0.0176 (7)	0.0226 (8)	0.0135 (7)	0.0112 (7)	0.0055 (6)
C15	0.0212 (8)	0.0136 (7)	0.0184 (8)	0.0059 (6)	0.0071 (6)	0.0013 (6)
C16	0.0182 (8)	0.0198 (8)	0.0246 (9)	0.0089 (7)	0.0097 (7)	0.0052 (7)
C17	0.0182 (8)	0.0163 (7)	0.0229 (8)	0.0082 (6)	0.0063 (7)	0.0042 (6)
C18	0.0301 (9)	0.0217 (8)	0.0292 (9)	0.0142 (7)	0.0165 (8)	0.0109 (7)
C19	0.0138 (7)	0.0133 (7)	0.0163 (7)	0.0050 (6)	0.0056 (6)	0.0012 (6)
C20	0.0201 (8)	0.0176 (7)	0.0220 (8)	0.0088 (6)	0.0096 (7)	0.0058 (6)
C21	0.0212 (8)	0.0238 (8)	0.0224 (9)	0.0050 (7)	0.0119 (7)	0.0029 (7)
C22	0.0177 (8)	0.0249 (9)	0.0282 (9)	0.0077 (7)	0.0109 (7)	−0.0037 (7)
C23	0.0201 (8)	0.0224 (8)	0.0289 (9)	0.0138 (7)	0.0081 (7)	0.0016 (7)
C24	0.0185 (8)	0.0165 (7)	0.0203 (8)	0.0086 (6)	0.0062 (6)	0.0030 (6)
C25	0.0215 (8)	0.0218 (8)	0.0173 (8)	0.0116 (7)	0.0073 (7)	0.0018 (6)
C26	0.0212 (8)	0.0201 (8)	0.0157 (7)	0.0120 (7)	0.0090 (6)	0.0002 (6)
C27	0.0253 (8)	0.0210 (8)	0.0200 (8)	0.0126 (7)	0.0113 (7)	0.0053 (7)
C28	0.0229 (8)	0.0221 (8)	0.0231 (9)	0.0070 (7)	0.0118 (7)	0.0024 (7)
C29	0.0191 (8)	0.0304 (9)	0.0229 (9)	0.0123 (7)	0.0075 (7)	0.0019 (7)
C30	0.0296 (9)	0.0260 (9)	0.0277 (9)	0.0198 (8)	0.0124 (8)	0.0082 (7)
C31	0.0243 (8)	0.0183 (8)	0.0245 (9)	0.0114 (7)	0.0124 (7)	0.0030 (7)

### Geometric parameters (Å, °)

**Table:**

S1—O1	1.4360 (12)	C14—C15	1.395 (2)
S1—O2	1.4401 (12)	C14—H14	0.93
S1—N1	1.6277 (13)	C15—C16	1.393 (2)
S1—C12	1.7649 (17)	C15—C18	1.507 (2)
N1—C11	1.4934 (19)	C16—C17	1.387 (2)
N1—C1	1.4996 (19)	C16—H16	0.93
N2—C9	1.406 (2)	C17—H17	0.93
N2—C10	1.448 (2)	C18—H18A	0.96
N2—H1N2	0.83 (2)	C18—H18B	0.96
C1—C2	1.526 (2)	C18—H18C	0.96
C1—H1A	0.97	C19—C24	1.393 (2)
C1—H1B	0.97	C19—C20	1.399 (2)
C2—C10	1.521 (2)	C20—C21	1.385 (2)
C2—C3	1.531 (2)	C20—H20	0.93
C2—H2	0.98	C21—C22	1.390 (3)
C3—C19	1.518 (2)	C21—H21	0.93
C3—C4	1.534 (2)	C22—C23	1.377 (3)
C3—H3	0.98	C22—H22	0.93
C4—C5	1.397 (2)	C23—C24	1.395 (2)
C4—C9	1.409 (2)	C23—H23	0.93
C5—C6	1.384 (2)	C24—H24	0.93
C5—H5	0.93	C25—C26	1.510 (2)
C6—C7	1.390 (2)	C25—H25A	0.97
C6—H6	0.93	C25—H25B	0.97
C7—C8	1.380 (2)	C26—C27	1.393 (2)
C7—H7	0.93	C26—C31	1.397 (2)
C8—C9	1.402 (2)	C27—C28	1.383 (2)
C8—H8	0.93	C27—H27	0.93
C10—C11	1.530 (2)	C28—C29	1.383 (3)
C10—H10	0.98	C28—H28	0.93
C11—C25	1.534 (2)	C29—C30	1.386 (3)
C11—H11	0.98	C29—H29	0.93
C12—C17	1.389 (2)	C30—C31	1.387 (2)
C12—C13	1.402 (2)	C30—H30	0.93
C13—C14	1.387 (2)	C31—H31	0.93
C13—H13	0.93		
			
O1—S1—O2	119.68 (7)	C14—C13—C12	118.98 (15)
O1—S1—N1	106.27 (7)	C14—C13—H13	120.5
O2—S1—N1	106.96 (7)	C12—C13—H13	120.5
O1—S1—C12	107.58 (8)	C13—C14—C15	121.68 (15)
O2—S1—C12	107.09 (7)	C13—C14—H14	119.2
N1—S1—C12	108.93 (7)	C15—C14—H14	119.2
C11—N1—C1	111.99 (12)	C16—C15—C14	118.02 (16)
C11—N1—S1	120.85 (10)	C16—C15—C18	120.34 (15)
C1—N1—S1	118.57 (10)	C14—C15—C18	121.62 (15)
C9—N2—C10	114.33 (12)	C17—C16—C15	121.59 (16)
C9—N2—H1N2	111.4 (14)	C17—C16—H16	119.2
C10—N2—H1N2	116.0 (14)	C15—C16—H16	119.2
N1—C1—C2	103.14 (12)	C16—C17—C12	119.40 (15)
N1—C1—H1A	111.1	C16—C17—H17	120.3
C2—C1—H1A	111.1	C12—C17—H17	120.3
N1—C1—H1B	111.1	C15—C18—H18A	109.5
C2—C1—H1B	111.1	C15—C18—H18B	109.5
H1A—C1—H1B	109.1	H18A—C18—H18B	109.5
C10—C2—C1	103.00 (12)	C15—C18—H18C	109.5
C10—C2—C3	109.13 (12)	H18A—C18—H18C	109.5
C1—C2—C3	118.14 (13)	H18B—C18—H18C	109.5
C10—C2—H2	108.7	C24—C19—C20	118.41 (15)
C1—C2—H2	108.7	C24—C19—C3	120.85 (14)
C3—C2—H2	108.7	C20—C19—C3	120.67 (14)
C19—C3—C2	113.47 (12)	C21—C20—C19	120.97 (16)
C19—C3—C4	111.61 (12)	C21—C20—H20	119.5
C2—C3—C4	108.54 (12)	C19—C20—H20	119.5
C19—C3—H3	107.7	C20—C21—C22	120.01 (17)
C2—C3—H3	107.7	C20—C21—H21	120.0
C4—C3—H3	107.7	C22—C21—H21	120.0
C5—C4—C9	117.86 (14)	C23—C22—C21	119.69 (16)
C5—C4—C3	120.29 (13)	C23—C22—H22	120.2
C9—C4—C3	121.85 (13)	C21—C22—H22	120.2
C6—C5—C4	122.35 (15)	C22—C23—C24	120.49 (16)
C6—C5—H5	118.8	C22—C23—H23	119.8
C4—C5—H5	118.8	C24—C23—H23	119.8
C5—C6—C7	119.10 (15)	C19—C24—C23	120.42 (16)
C5—C6—H6	120.4	C19—C24—H24	119.8
C7—C6—H6	120.4	C23—C24—H24	119.8
C8—C7—C6	120.19 (15)	C26—C25—C11	110.75 (13)
C8—C7—H7	119.9	C26—C25—H25A	109.5
C6—C7—H7	119.9	C11—C25—H25A	109.5
C7—C8—C9	120.77 (15)	C26—C25—H25B	109.5
C7—C8—H8	119.6	C11—C25—H25B	109.5
C9—C8—H8	119.6	H25A—C25—H25B	108.1
C8—C9—N2	119.04 (14)	C27—C26—C31	118.39 (15)
C8—C9—C4	119.73 (14)	C27—C26—C25	120.46 (15)
N2—C9—C4	121.22 (14)	C31—C26—C25	120.94 (15)
N2—C10—C2	107.97 (13)	C28—C27—C26	120.86 (16)
N2—C10—C11	115.04 (12)	C28—C27—H27	119.6
C2—C10—C11	104.70 (12)	C26—C27—H27	119.6
N2—C10—H10	109.6	C27—C28—C29	120.38 (16)
C2—C10—H10	109.6	C27—C28—H28	119.8
C11—C10—H10	109.6	C29—C28—H28	119.8
N1—C11—C10	100.91 (11)	C28—C29—C30	119.44 (16)
N1—C11—C25	112.14 (13)	C28—C29—H29	120.3
C10—C11—C25	113.02 (13)	C30—C29—H29	120.3
N1—C11—H11	110.2	C29—C30—C31	120.37 (17)
C10—C11—H11	110.2	C29—C30—H30	119.8
C25—C11—H11	110.2	C31—C30—H30	119.8
C17—C12—C13	120.33 (15)	C30—C31—C26	120.50 (16)
C17—C12—S1	120.10 (13)	C30—C31—H31	119.8
C13—C12—S1	119.54 (13)	C26—C31—H31	119.8
			
O1—S1—N1—C11	−169.30 (12)	N2—C10—C11—C25	−86.66 (17)
O2—S1—N1—C11	−40.37 (14)	C2—C10—C11—C25	155.01 (13)
C12—S1—N1—C11	75.06 (13)	O1—S1—C12—C17	−16.92 (15)
O1—S1—N1—C1	45.43 (13)	O2—S1—C12—C17	−146.77 (13)
O2—S1—N1—C1	174.35 (12)	N1—S1—C12—C17	97.88 (14)
C12—S1—N1—C1	−70.22 (13)	O1—S1—C12—C13	160.95 (12)
C11—N1—C1—C2	−7.78 (16)	O2—S1—C12—C13	31.10 (14)
S1—N1—C1—C2	140.39 (11)	N1—S1—C12—C13	−84.25 (13)
N1—C1—C2—C10	29.34 (15)	C17—C12—C13—C14	−0.2 (2)
N1—C1—C2—C3	149.66 (13)	S1—C12—C13—C14	−178.03 (12)
C10—C2—C3—C19	−172.66 (13)	C12—C13—C14—C15	0.3 (2)
C1—C2—C3—C19	70.25 (18)	C13—C14—C15—C16	0.1 (2)
C10—C2—C3—C4	−47.97 (16)	C13—C14—C15—C18	−178.40 (15)
C1—C2—C3—C4	−165.07 (13)	C14—C15—C16—C17	−0.7 (2)
C19—C3—C4—C5	−38.87 (19)	C18—C15—C16—C17	177.88 (15)
C2—C3—C4—C5	−164.65 (14)	C15—C16—C17—C12	0.8 (2)
C19—C3—C4—C9	141.90 (15)	C13—C12—C17—C16	−0.4 (2)
C2—C3—C4—C9	16.12 (19)	S1—C12—C17—C16	177.50 (12)
C9—C4—C5—C6	0.7 (2)	C2—C3—C19—C24	−113.47 (16)
C3—C4—C5—C6	−178.57 (15)	C4—C3—C19—C24	123.52 (15)
C4—C5—C6—C7	−0.5 (3)	C2—C3—C19—C20	69.60 (18)
C5—C6—C7—C8	−0.1 (3)	C4—C3—C19—C20	−53.42 (18)
C6—C7—C8—C9	0.6 (3)	C24—C19—C20—C21	−0.5 (2)
C7—C8—C9—N2	−179.10 (15)	C3—C19—C20—C21	176.54 (14)
C7—C8—C9—C4	−0.4 (2)	C19—C20—C21—C22	0.4 (2)
C10—N2—C9—C8	−159.03 (14)	C20—C21—C22—C23	−0.4 (3)
C10—N2—C9—C4	22.3 (2)	C21—C22—C23—C24	0.6 (3)
C5—C4—C9—C8	−0.2 (2)	C20—C19—C24—C23	0.7 (2)
C3—C4—C9—C8	179.05 (14)	C3—C19—C24—C23	−176.33 (14)
C5—C4—C9—N2	178.46 (15)	C22—C23—C24—C19	−0.8 (2)
C3—C4—C9—N2	−2.3 (2)	N1—C11—C25—C26	167.76 (13)
C9—N2—C10—C2	−55.04 (17)	C10—C11—C25—C26	54.52 (18)
C9—N2—C10—C11	−171.53 (13)	C11—C25—C26—C27	−102.25 (18)
C1—C2—C10—N2	−164.09 (12)	C11—C25—C26—C31	72.43 (19)
C3—C2—C10—N2	69.58 (15)	C31—C26—C27—C28	0.4 (2)
C1—C2—C10—C11	−41.05 (15)	C25—C26—C27—C28	175.20 (15)
C3—C2—C10—C11	−167.38 (12)	C26—C27—C28—C29	−2.0 (3)
C1—N1—C11—C10	−16.78 (16)	C27—C28—C29—C30	1.4 (3)
S1—N1—C11—C10	−164.12 (11)	C28—C29—C30—C31	0.9 (3)
C1—N1—C11—C25	−137.32 (14)	C29—C30—C31—C26	−2.5 (3)
S1—N1—C11—C25	75.33 (16)	C27—C26—C31—C30	1.9 (2)
N2—C10—C11—N1	153.42 (13)	C25—C26—C31—C30	−172.91 (15)
C2—C10—C11—N1	35.09 (15)		

### Hydrogen-bond geometry (Å, °)

**Table:**

*D*—H···*A*	*D*—H	H···*A*	*D*···*A*	*D*—H···*A*
N2—H1N2···Cg2	0.83 (2)	2.61 (2)	3.374 (2)	152 (2)
C29—H29···Cg1^i^	0.93	2.90	3.605 (2)	134

Symmetry codes: (i) *x*+1, *y*, *z*.
